# Low 25-Hydroxyvitamin D Levels Are Associated with Infections and Mortality in Patients with Cirrhosis

**DOI:** 10.1371/journal.pone.0132119

**Published:** 2015-06-29

**Authors:** Fabian Finkelmeier, Bernd Kronenberger, Stefan Zeuzem, Albrecht Piiper, Oliver Waidmann

**Affiliations:** Medizinische Klinik 1, Schwerpunkt Gastroenterologie und Hepatologie, Universitätsklinikum Frankfurt, Goethe-Universität, Theodor-Stern-Kai 7, D-60590 Frankfurt/Main, Germany; University of Navarra School of Medicine and Center for Applied Medical Research (CIMA), SPAIN

## Abstract

**Background & Aims:**

Vitamin D, best known to regulate bone mineralization, has numerous additional roles including regulation inflammatory pathways. Recently, an increased incidence of 25-hydroxyvitamin D3 (25(OH)D_3_) deficiency has been found in subjects suffering from liver diseases. We here investigated if low vitamin D levels might be associated with prognosis, inflammation and infectious complications in patients with cirrhosis.

**Methods:**

We performed a prospective cohort study investigating the relation between 25(OH)D_3_ levels and stages of cirrhosis, mortality and complications of cirrhosis, including infections.

**Results:**

251 patients with cirrhosis were enrolled into the present prospective cohort study. 25(OH)D_3_ levels were quantified by radioimmunoassay from serum samples obtained at study inclusion. The mean follow-up time was 411 ± 397 days with a range of 1-1382 days. 30 (12.0%) patients underwent liver transplantation and 85 (33.8%) individuals died within the study. The mean serum 25(OH)D_3_ concentration was 8.93 ± 7.1 ng/ml with a range of 1.0 to 46.0 ng/ml. 25(OH)D_3_ levels differed significantly between Child Pugh scores and showed a negative correlation with the model of end stage liver disease (MELD) score. Patients with decompensated cirrhosis and infectious complications, had significantly lower 25(OH)D_3_ levels compared to subjects without complications. Low 25(OH)D_3_ was associated with mortality in uni- as well as multivariate Cox regression models.

**Conclusions:**

25(OH)D_3_ deficiency is associated with advanced liver disease and low 25(OH)D_3_ levels are an indicator for a poor outcome and are associated with infectious complications.

## Introduction

Cirrhosis is a consequence of chronic liver diseases, characterized by liver injury, chronic inflammation and finally by progressive substitution of liver parenchyma by scar tissue. Cirrhosis results in progressive liver failure and is accompanied by various complications such as portal hypertension, ascites, gastrointestinal bleeding or hepatocellular carcinoma (HCC) [1]. It accounts for about 2% of all deaths in Europe (170.000/year) with increasing mortality rates in several countries [2]. Alcohol abuse and infections with hepatotropic viruses, especially hepatitis C virus (HCV) infection, have been the predominant risk factors for cirrhosis in Western countries within the last decades. However, the prevalence of non-alcoholic fatty liver disease (NAFLD) has risen dramatically, affecting up to 40% of all Europeans and more than 50% of patients suffering of type 2 diabetes within the last years [3]. Patients with NAFLD are at risk to develop non-alcoholic steatohepatitis (NASH) which is supposed to become the major cause of cirrhosis within the following decades [4]. The prognosis of cirrhotic individuals is determined by the occurrence of hepatic complications [3]. Patients with compensated cirrhosis without clinically evident portal hypertension have a relatively favorable prognosis. On the other hand, patients with decompensated disease, who suffer from ascites as well as variceal bleeding have one year mortality rates of more than 50% [3]. Commonly used predictors of prognosis are the Child-Pugh-Score [4] and the model for end stage liver disease (MELD) [5].

Vitamin D is best known for its function in calcium homeostasis and bone mineralization [6]. However, it has numerous additional roles such as regulation of proliferation, apoptosis, differentiation and inflammation [7,8]. Vitamin D generation is a multi-step process involving the skin, the liver and the kidneys. Cholecalciferol is hydroxylated to the bioactive 25-hydroxyvitamin D3 (25(OH)D_3_) in the liver and is bound to the vitamin-D-binding-protein (DBP) [8]. 1α-hydroxylase converts 25(OH)D_3_ to 1α,25-dihydroxyvitamin D3 (1,25(OH)D_3_) mainly in the kidneys. 1,25(OH)D_3_, known as calcitriol, is the most bioactive form [8]. Recently, an increased incidence of 25(OH)D_3_ deficiency has been found in individuals suffering from liver diseases and the severity of 25(OH)D_3_ deficiency in the patients correlated with the severity of liver dysfunction [9,10,11]. Liver disease is accompanied by activation of the innate immune system and vitamin D levels inversely correlate with the expression of toll like receptors (TLRs) in monocytes, indicating an inverse correlation between vitamin D levels and systemic inflammation [9]. Strong inflammatory conditions displayed by high levels of C-reactive protein (CRP) or soluble CD163 (sCD163) are associated with an unfavorable prognosis in patients with cirrhosis [12,13]. Observations in cirrhotic patients showed a poor prognosis in individuals with low 25(OH)D_3_ levels [14,15]. Given the fact that cirrhotic patients presenting with infections or elevated inflammatory conditions indicated by high levels of CRP or sCD163, have an unfavorable prognosis [12,13,16] and that expression of TLRs inversely correlate with vitamin D levels [9], we hypothesized that low vitamin D levels might be associated with inflammatory responses and infectious complications in cirrhotic patients. Therefore, we performed a prospective cohort study investigating the relation of 25(OH)D_3_ levels with stages of cirrhosis and mortality as well as the association of 25(OH)D_3_ concentrations and complications of cirrhosis, including infectious complications.

## Patients and Methods

### Selection of patients

Between May 2009 and June 2011 patients with cirrhosis presenting at the Department of Internal Medicine 1 of the Frankfurt University Hospital were consecutively enrolled into the present prospective cohort study. All patients gave their written informed consent prior to study inclusion. The study population has been described before [13].

The inclusion criteria were cirrhosis assessed by liver histopathological examination or pathognomonic results in ultrasound, computed tomography (CT) or magnetic resonance imaging (MRI). Exclusion criteria were patients below 18 years of age, a history of cancer other than hepatocellular carcinoma within the last five years or a history of solid organ transplantation. Furthermore, patients with cholestatic liver diseases, namely primary biliary cirrhosis (PBC) and primary sclerosing cholangitis (PSC) were excluded from the analysis. Patients who were eligible for liver transplantation were listed for liver transplantation. Organ allocation was performed by Eurotransplant according to Eurotransplant and German guidelines. Patients were included in the study from the day of written informed consent and were followed-up until death, liver transplantation or last contact. Patients who underwent liver transplantation were excluded from further analysis from the day of transplantation. In patients who presented with clinical signs of infections (fever, chills, coughing or dysuria) or showed elevated CRP or leukocyte values in the blood tests, urinalysis and x-ray of the chest were performed. If urinalysis showed presence of leucocytes, urine cultures were initiated. In patients who had clinical or radiological signs of lung infections sputum cultures were done. In severely sick patients with pneumonia bronchoalveolar lavage was performed to gain material for cultures. If diarrhea was reported in patients with suspected infection, stool cultures were initiated. If ascites was present paracentesis was mandatory to rule out SBP.

Child-Pugh [3] and MELD [4] and scores were assessed by clinical examination, laboratory parameters and results of abdominal ultrasound examination, CT or MRI at the time of inclusion in the study. Diagnosis of infection was based on clinical presentation and laboratory results. Diagnosis and treatment of ascites with or without spontaneous bacterial peritonitis were performed according to EASL guidelines [17].

The study was performed in accordance with the 1975 Declaration of Helsinki and the REMARK guidelines [18] for prospective biomarker studies. The study was approved by the institutional review board of the Frankfurt University Hospital.

### Blood sampling

Blood sampling and storage of samples was performed as described before [13]. Routine laboratory parameters were determined at the Central Laboratory of the Frankfurt University Hospital.

### Vitamin D measurements

25(OH)D_3_ levels were quantified by radioimmunoassay (I125 Radioimmunoassay IA Kit; DiaSorin, Stillwater, MN) on a Multi Crystal LB2111 Gamma Counter (Berthold, Bad Wildbad, Germany) from serum samples obtained at the day of study inclusion as described recently [19]. Samples were blinded for the person who performed measurements, and all measurements were determined at the same day from prospectively collected and stored serum samples. Serum 25(OH)D_3_ concentrations <10 ng/ml were defined as severe vitamin D deficiency; levels from 10 to 20 ng/ml were considered as insufficiency and serum levels >20 ng/ml were considered normal. Serum soluble CD163 (sCD163) concentrations were assessed with the Macro163 sandwich enzyme-linked immunosorbent assay (ELISA) (Trillium Diagnostics, Bangor, ME) according to the recommendation of the manufacturer as described previously [13]. Samples were measured in duplicates on a Tecan SLT Rainbow plate reader (Tecan, Männedorf, Switzerland).

### Statistical analysis

This study was designed as a prospective cohort study. Patients were included on the day of presentation after giving written informed consent. They were followed until death or last contact. The primary end point was overall survival. Predictors of survival were determined using a univariate Cox regression hazard model. Death was recorded as event. For assessment of independent predictors of survival a multivariate Cox regression hazard model with forward stepwise (likelihood ratio) entry was used. Survival curves with the estimated hazards were calculated with the Cox regression model. Statistical analyses were performed with SPSS (Version 22.0, IBM, New York, USA) and BiAS (Version 10.03, Epsilon-Verlag, Darmstadt, Germany). Continuous variables are shown as means ± standard deviation and categorical variables are reported as frequencies and percentages. Differences in the serum biomarker values between different patient cohorts were determined using the nonparametric Wilcoxon-Mann-Whitney and Kruskal-Wallis tests. For sub-analysis of a statistically significant Kruskal-Wallis test, the Bonferroni correction was used. P values < 0.05 were considered to be significant. In the box plots the vertical lines indicate the range, the horizontal boundaries of the boxes represent the first and third quartile. The correlation coefficient r between different parameters was calculated by using the Spearman correlation.

## Results

251 patients with cirrhosis were included in the present study. Patients´ characteristics are summarized in Table 1. In the predominant number of cases cirrhosis was a result of alcohol abuse, hepatitis B virus (HBV) or HCV infection. 46 patients (18.3%) concurrently suffered from HCC. 192 patients (76.5%) showed complications of cirrhosis (ascites, spontaneous bacterial peritonitis (SBP), gastrointestinal bleeding, hepatorenal syndrome (HRS) or hepatic encephalopathy) at the day of inclusion in the study, whereas 59 patients (23.5%) had compensated disease. The mean follow-up time was 411 +/- 397 days with a range of 1–1382 days. 30 patients (12.0%) underwent liver transplantation within the observation time after study inclusion and were excluded from further analysis from the day of transplantation. 85 individuals (33.8%) died within the study. The main reasons of death were liver failure, sepsis with concomitant multi organ failure, and variceal bleeding.

**Table 1:** Patient characteristics.

Parameter	Patients
**Epidemiology**	
Patients n	251
Gender, m/f (%)	171/80 (68.1/31.9)
Age, median, range	57, 25–84
**Etiology of liver disease**	
Alcohol abuse, n (%)	135 (55.8%)
Hepatitis C, n (%)	74 (29.5%)
Hepatitis B, n (%)	34 (13.5%)
NASH^1^, n (%)	7 (2.8%)
AIH, n (%)	4 (1.6%)
Hemochromatosis, n (%)	7 (2.6%)
Cryptogenic, n (%)	23 (9.2%)
**HCC**	
Diagnosed HCC	46 (18.3%)
**Child-Pugh stage**	
A, n (%)	51 (20.3%)
B, n (%)	118 (47.0%)
C, n (%)	82 (32.7%)
**MELD** ^**2**^, median, range	15 (6–40)
**Treatment**	
Liver transplantation, n (%)	30 (12.0%)
**Laboratory results**	
Sodium (mmol/l), median, range	138, 111–150
ALT^3^ (U/l), median, range	32, 2–1594
AST^4^ (U/l), median, range	53, 15–2823
GGT^5^ (U/l), median, range	104, 14–1178
ALP^6^ (U/l), median, range	116, 31–688
Bilirubin (mg/dl), median, range	2.0, 0.2–26.8
Albumin (mg/dl), median, range	3.2, 1.6–5.2
INR^7^, mean, median, range	1.41, 0.89–4.2
Creatinine (mg/dl), median, range	1.02, 0.38–6.77
CRP^8^ (mg/dl), median, range	1.19, 0.03–16.84
Hb (mg/dl), median, range	10.5, 6.0–18.0
HbA1c (%), median, range	5.3, 3.7–9.8
Thrombocytes (x10^9^/L)	98, 17–1507
Leukocytes/nl	5.23, 0.63–56.63

Abbreviations:

^1^NASH, non-alcoholic steatohepatitis;

^2^MELD, model of end stage liver disease;

^3^ALT, alanine aminotransferase,

^4^AST, aspartate aminotransferase;

^5^GGT, gamma-glutaryl-transferase;

^6^ALP, alkaline phosphatase;

^7^INR, internationalized ratio;

^8^CRP, C-reactive protein.

### 25(OH)D_3_ levels among different etiologies of chronic liver disease

The mean serum 25(OH)D_3_ concentration was 8.9 ± 7.1 ng/ml with a range of 1.0 to 46.0 ng/ml. 173 patients (68.9%) had very low 25(OH)D_3_ levels (< 10 ng/ml), 59 patients (23.5%) had low levels (10–20 ng/ml) and 19 (7.6%) patients had normal 25(OH)D_3_ levels (> 20 ng/ml).

**Fig 1:**
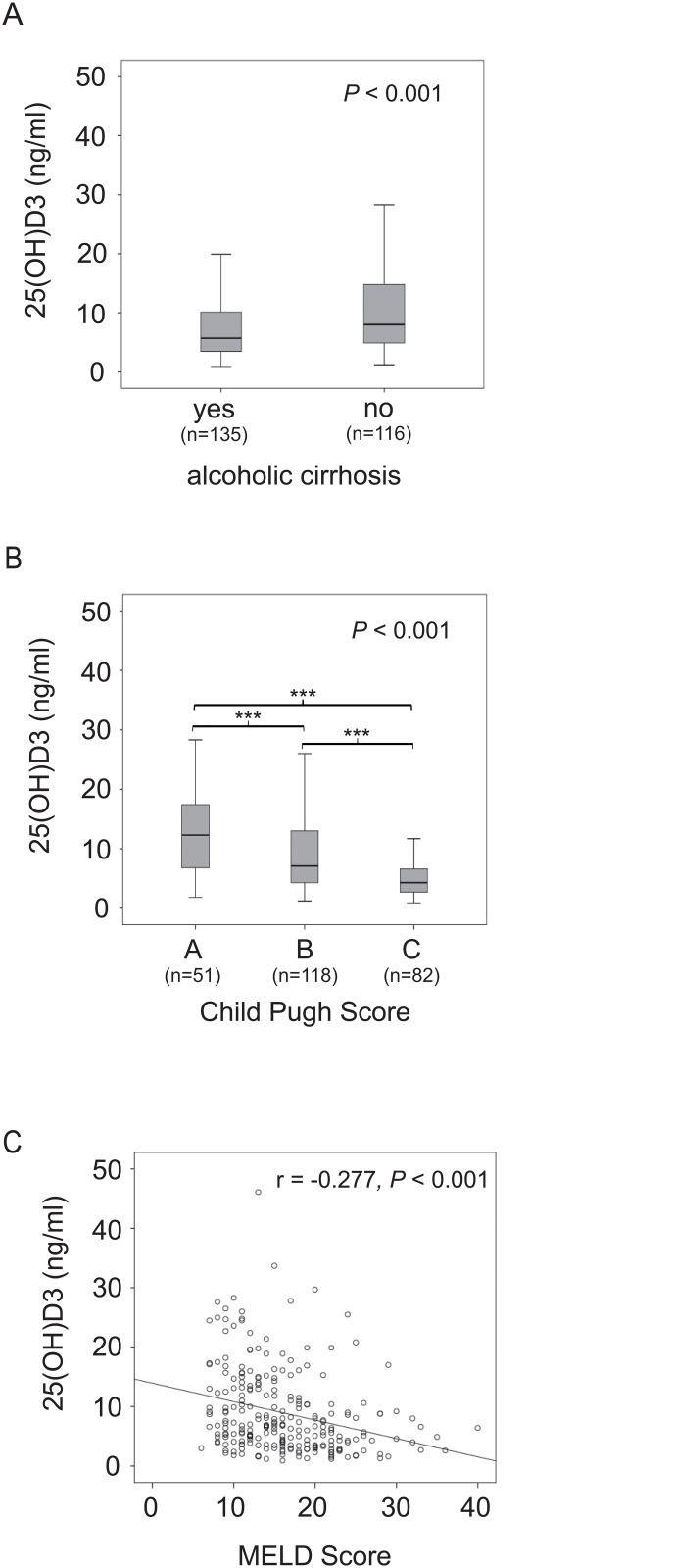
Association of Vitamin D levels with etiologies and stages of cirrhosis. Vitamin D levels in patients with and without alcoholic liver disease (**1A**). Serum 25(OH)D_3_ concentrations in stages of cirrhosis (**1B**) and its correlation with the MELD score (**1C**).

44 patients (17.5%) received oral vitamin D supplementation at the day of study enrollment. Of the 44 patients, 30 patients received 1000 IU or less 25(OH)D_3_ per day and six subjects took more than 1000 IU 25(OH)D_3_ daily. Eight individuals were supplemented with 250 μg 1α,25(OH)_2_D_3_ per day. One of the patients taking 250 μg 1α,25(OH)_2_D_3_ per day was also on 25(OH)D_3_ supplementation (1000 IU per day). The mean serum 25(OH)D_3_ concentration in subjects with vitamin D supplementation was significantly higher (12.4 ± 9.6 ng/ml) compared to individuals without supplementation (8.2 ± 6.2 ng/ml) (*P* = 0.003). As the main etiologies of cirrhosis were alcohol abuse and infections with HBV or HCV, 25(OH)D_3_ levels were compared between patients with and without alcoholic liver disease, HBV or HCV infections. 25(OH)D_3_ levels did not significantly differ between patients with or without HBV or HCV infection (*P* = 0.117 and *P* = 0.092, respectively). However, patients with alcoholic liver cirrhosis had significantly lower serum 25(OH)D_3_ concentrations compared to individuals with other causes of cirrhosis (*P* = 0.001) (Fig 1A). There was no significant difference in 25(OH)D_3_ levels in male compared to female patients (*P* = 0.311). Serum 25(OH)D_3_ levels did not significantly differ between blood samples acquired during summer/spring or winter/autumn time (*P* = 0.369).

### 25(OH)D_3_ levels differ among stages of cirrhosis

It has been shown that 25(OH)D_3_ levels decrease with the progress of cirrhosis. Therefore, 25(OH)D_3_ levels were compared between stages of cirrhosis. As shown in Fig 1B, there were significant differences among Child Pugh scores with the highest levels in Child A and the lowest levels in Child C patients (*P* < 0.001). In addition, the relation between 25(OH)D_3_ levels and the MELD score was assessed. There was a significant inverse correlation between 25(OH)D_3_ concentrations and the MELD score (r = -0.277, *P* < 0.001) (Fig 1C), indicating a relation between severity of cirrhosis and low 25(OH)D_3_ levels. 192 patients had decompensated cirrhosis at the day of study inclusion coming along with portal hypertension, ascites, hepatic encephalopathy or varices. Ascites and HRS are typical complications of liver insufficiency and portal hypertension, indeed patients without or with moderate ascites had significantly higher 25(OH)D_3_ levels compared to individuals with massive ascites (*P* < 0.001). Individuals with HRS, a severe complication of cirrhosis, had significantly lower 25(OH)D_3_ levels in contrast to subjects without HRS (*P* < 0.001) (**Data not shown**).

### 25(OH)D_3_ and infectious complications

**Fig 2:**
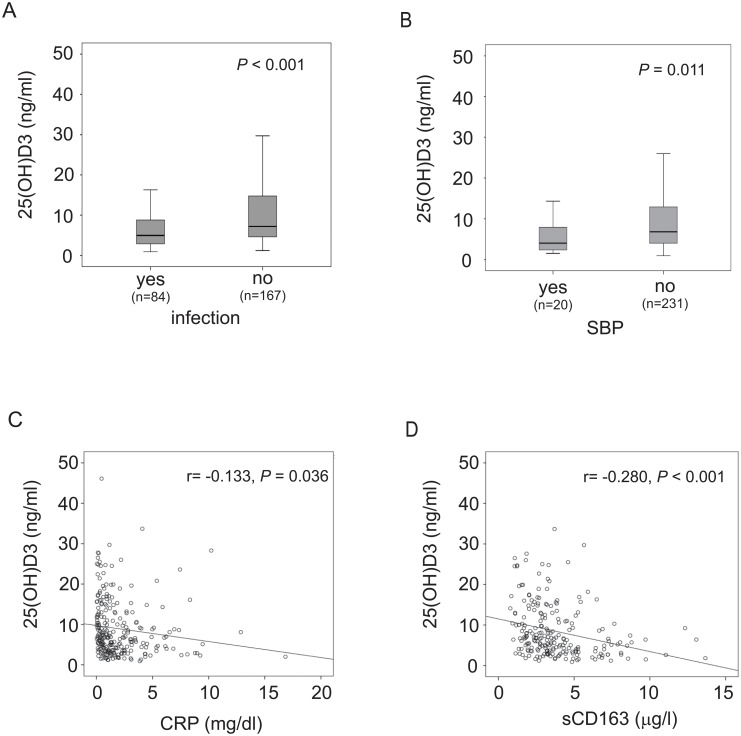
Relation of 25(OH)D_3_ levels and infections. Serum Vitamin D levels in patients with and without infections (**2A**) and spontaneous bacterial peritonitis (**2B**). Relations of 25(OH)D_3_ concentrations and C-reactive protein (CRP) levels (**2C**) as well as soluble CD163 (sCD163) concentrations (**2D**) are shown.

Bacterial infection is a frequent cause of hepatic decompensation. 84 of the 251 patients (33.5%) presented with bacterial infections on the day of study inclusion, namely SBP (n = 20), urinary tract infections (n = 41), pulmonary infections (n = 16) and other infections (n = 7). Patients suffering from infections at the day of study inclusion had significantly lower 25(OH)D_3_ levels compared to individuals without infections (P < 0.001) (Fig 2A). SBP is a severe infectious complication with a high mortality among cirrhotics. Patients with SBP had significantly lower 25(OH)D_3_ levels than patients without SBP (*P* = 0.011) (Fig 2B). As vitamin D levels were lower in patients with clinical apparent infections and were reported to be inversely correlated with the activation of the innate immune system in chronic liver diseases (9), the relationship of the surrogate parameters for systemic inflammation, namely CRP and sCD163, were compared to 25(OH)D_3_ levels. As shown in Fig 2C and 2D, 25(OH)D_3_ levels correlated inversely with CRP (r = -0.133, *P* = 0.036) and sCD163 levels (r = -0.280, *P* < 0.001).

### Low 25(OH)D_3_ levels are an independent risk factor for mortality in cirrhotics

**Fig 3:**
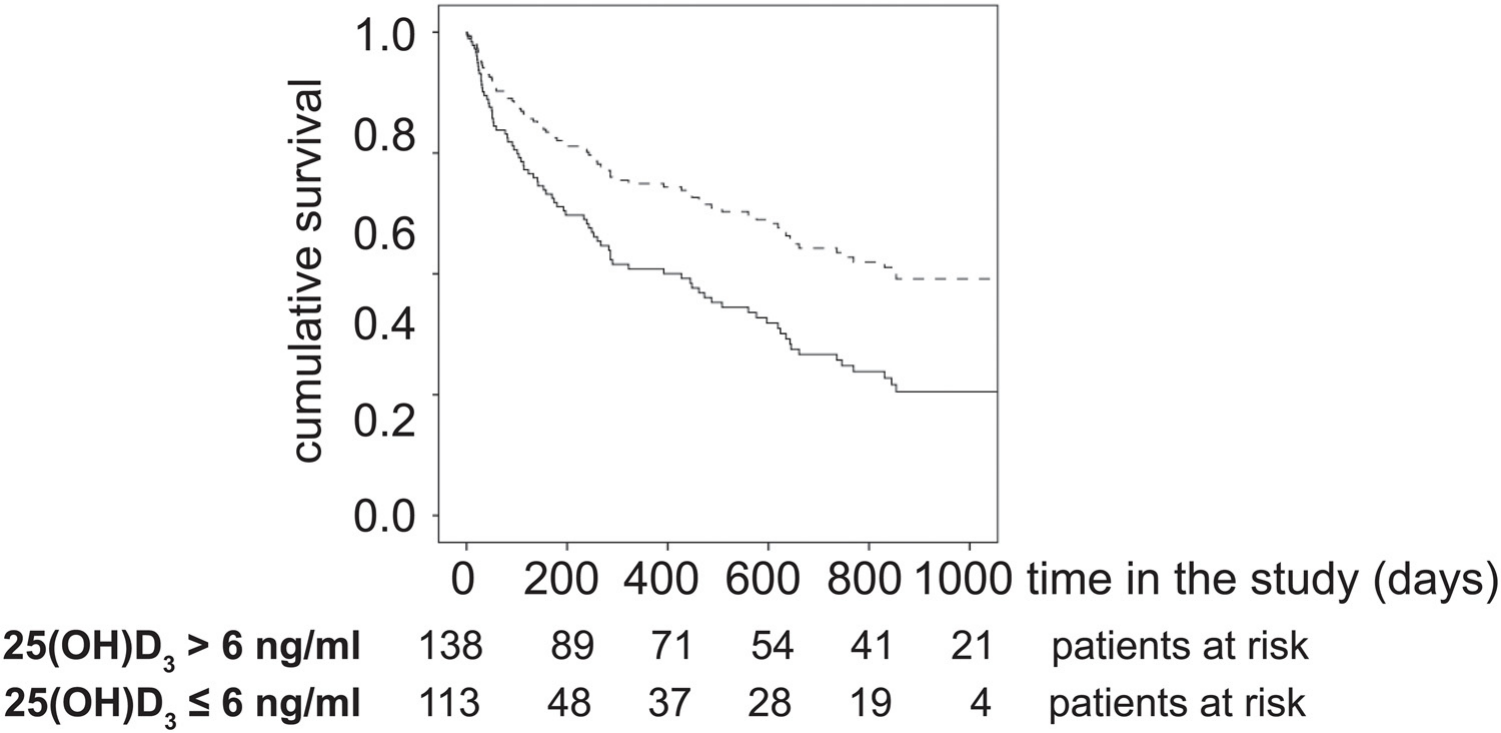
Relation of Vitamin D concentrations and mortality. The overall survival times of patients with the indicated Vitamin D levels are shown.

As patients with advanced cirrhosis had lower serum 25(OH)D_3_ concentrations, we hypothesized that 25(OH)D_3_ levels might be of prognostic value in these patients. Recently, serum 25(OH)D_3_ concentrations of 6 ng/ml or 10 ng/ml indicating 25(OH)D_3_ deficiency have been proposed to be of prognostic value in cirrhotic individuals with or without HCC, respectively [14,15,19]. 25(OH)D_3_ levels ≤ 6 ng/ml were associated with higher mortality compared to higher 25(OH)D_3_ levels in the present cohort (HR 1.723, 95% CI 1.122–2.646, *P* = 0.013 for 6 ng/ml). However, serum 25(OH)D_3_ concentrations ≤ 10 ng/ml were not significantly associated with higher mortality HR 1.424, 95% CI 0.889–2.279, *P* = 0.141 for 10 ng/ml) (Fig 3). Several patients received vitamin D substitution. Therefore, the impact of vitamin D alimentation was investigated. Substitution with vitamin D was not significantly associated with overall survival (HR 0.841, 95% CI 0.473–1.494, *P* = 0.555).

To further analyze if serum 25(OH)D_3_ level might be an independent prognostic parameter a multivariate Cox regression model with forward stepwise likelihood ratio was performed. The nominal variables age (≤ 57 years vs. > 57 years), gender, HCC, serum sCD163 levels (≤ 3950 ng/l vs. > 3950 ng/l), presence of infection, Child Pugh score (A,B,C) as well as the continuous variables MELD score and 25(OH)D_3_ levels were included in the analysis. As shown in Table 2 25(OH)D_3_ levels < 6 ng/ml, HCC, infections and the MELD score were parameters independently associated with mortality.

**Table 2:** Univariate and multivariate analyses of parameters associated with mortality.

	Univariate analysis			Multivariate analysis		
Parameter	HR^1^	95% CI^2^	*P* value	HR	95% CI	*P* value
Male gender	1.407	0.902–2.196	0.133			
Age > 57 years	1.191	0.775–1.832	0.425			
MELD score^3^	1.098	1.063–1.135	< 0.001	1.096	1.055–1.140	< 0.001
Log 25(OH)D^3^	0.422	0.215–0.828	0.012			
25(OH)D^3^ < 6 ng/ml	1.723	1.122–2646	0.013	1.703	1.038–2.794	0.035
HCC^4^	2.187	1.328–3.602	0.002	3.399	1.762–6558	< 0.001
sCD163^5^ ≤ 3950ng/l	0.360	0.227–0572	< 0.001			
Infection	2.706	1.749–4.188	< 0.001	1.878	1.140–3.093	0.013
Child Pugh A vs. C	0.273	0.137–0.543	< 0.001			
Child Pugh B vs. C	0.606	0.381–0.963	0.034			

Abbreviations:

^1^HR, hazard ration;

^2^CI, confidence interval;

^3^MELD, model of end stage liver disease;

^4^HCC, hepatocellular carcinoma;

^5^sCD163, soluble CD163.

## Discussion

Vitamin D has manifold functions including bone homeostasis and regulation of immune responses. In the present study, we showed that serum 25(OH)D_3_ correlated with the severity of liver insufficiency and were associated with infectious complications. Furthermore, very low serum 25(OH)D_3_ concentrations were an independent risk factor for mortality in patients with cirrhosis in addition to the MELD score, HCC and infections.

Liver fibrosis and especially cirrhosis are linked to low 25(OH)D_3_ levels in alcoholic liver disease as well as in HCV related disease with and without HIV coinfection [14,20,21]. In accordance with previous reports patients with alcoholic related cirrhosis showed lower 25(OH)D_3_ levels compared to patients with other etiologies of advanced liver disease [14,22]. The reasons for particularly low vitamin levels in individuals with alcoholic cirrhosis are unknown. An alimentary deficit itself might be causative. We found in our cirrhotic patients a negative relation of liver function, namely MELD score and Child Pugh score, and serum vitamin D concentrations, which has also been observed in other cohorts of patients suffering from liver diseases [23]. Severe liver insufficiency is a reasonable cause for disturbance of the 25(OH)D_3_ generation. The hydroxylation of cholecalciferol to its bioactive form, namely 25(OH)D_3_, is carried out by the liver, showing its key role in vitamin D metabolism [8,9]. This hypothesis is supported by the lack of difference between the 25(OH)D_3_ levels determined either in spring/summer or autumn/winter, indicating that an insufficient hydroxylation of cholecalciferol rather than a lack of sun exposure might be the main reason of vitamin D deficiency in cirrhotic patients. An uncoupling of vitamin D levels and sun exposure has recently been found in patients suffering from HCC [19]. Furthermore, patients with decompensated cirrhosis had lower serum 25(OH)D_3_ levels compared to patients with compensated disease. Similarly to a previous report, patients with the severe complication of cirrhosis, SBP had significantly lower serum vitamin D [24]. Additionally, stressing the relation of vitamin D deficiency and advanced cirrhosis patients with complications of portal hypertension such as massive ascites and HRS had lower levels of serum 25(OH)D_3_ compared to individuals without the indicated complications.

A common cause of hepatic decompensation in cirrhotic patients is the occurrence of bacterial infections [25]. 25(OH)D_3_ modulates inflammatory responses and restrains innate immune responses [26,27], e.g. it limits the activation of monocytes and thereby reduces the production of pro-inflammatory cytokines including tumor necrosis factor α (TNFα) [27]. TNFα plays a critical role in the inflammatory environment and it is considered to be one of the main factors leading to cirrhosis [28]. Experimental studies support the idea of a suppressive effect of 25(OH)D_3_ on TNFα release [29–31]. A central pathogenic factor in cirrhosis is bacterial translocation from the gastrointestinal tract into the portal system, leading to sustained inflammatory responses and progression of liver disease. The translocated bacteria may also cause direct infectious complications. Vitamin D protects the gut barrier and prevents bacterial translocation [24,32,33]. Moreover, an increased portal hypertension is associated with lower 25(OH)D_3_ levels in patients with alcoholic liver disease [14]. In the present cohort of cirrhotic patients, serum vitamin D concentrations were not only inversely correlated with surrogate parameters of inflammation such as CRP or sCD163, but they were also lower in individuals with clinical apparent infections including SBP.

Low vitamin D levels do not only correlate with the extent of liver dysfunction but are also associated with a less favorable outcome [15,23,34,35]. Recent reports showed their association with higher mortality in alcoholic liver disease [14] as well as cirrhosis of other origins [15]. However, the published studies analyzed rather small cohorts of patients [15] or included only individuals with alcoholic liver disease [14]. Our results that 25(OH)D_3_ levels inversely correlated with the MELD score are in agreement with recent data [15,23] showing that serum 25(OH)D_3_ decreases with increasing degrees of liver insufficiency and therefore with prognosis. Though, we found in the present study that serum 25(OH)D_3_ was an independent risk factor for mortality in addition to the stage of cirrhosis, HCC and infectious complications. To our knowledge, our prospectively recruited cohort is the largest cirrhosis cohort published so far investigating the relation of serum vitamin D levels and overall survival in a prospective fashion.

In conclusion vitamin D is not only associated with the severity of liver disease and infections, but also an independent prognostic parameter. Whether 25(OH)D_3_ supplementation may improve prognosis of cirrhotic patients warrants prospective interventional trials.

## References

[1] Hernandez-Gea V, Friedman SL. Pathogenesis of liver fibrosis. Annu Rev Pathol 2011;6:425–456. 10.1146/annurev-pathol-011110-130246 21073339

[2] Blachier M, Leleu H, Peck-Radosavljevic M, Valla DC, Roudot-Thoraval F. The burden of liver disease in Europe: a review of available epidemiological data. J Hepatol 2013;58:593–608. 10.1016/j.jhep.2012.12.005 23419824

[3] D’Amico G, Garcia-Tsao G, Pagliaro L. Natural history and prognostic indicators of survival in cirrhosis: a systematic review of 118 studies. J Hepatol 2006;44:217–231. 16298014 10.1016/j.jhep.2005.10.013

[4] Child CG, Turcotte JG. Surgery and portal hypertension. Major Probl Clin Surg 1964;1:1–85. 4950264

[5] Kamath PS, Wiesner RH, Malinchoc M, Kremers W, Therneau TM, Kosberg CL, et al A model to predict survival in patients with end-stage liver disease. Hepatology 2001;33:464–470. 11172350 10.1053/jhep.2001.22172

[6] Heaney RP. The vitamin D requirement in health and disease. J Steroid Biochem Mol Biol 2005;97:3–19. 16026981 10.1016/j.jsbmb.2005.06.020

[7] Vanoirbeek E, Krishnan A, Eelen G, Verlinden L, Bouillon R, Feldman D, et al The anti-cancer and anti-inflammatory actions of 1,25 OH-D3. Best Pract Res Clin Endocrinol Metab 2011;25:593–604. 10.1016/j.beem.2011.05.001 21872801 PMC3164534

[8] Rosen JC. Vitamin D insufficiency. NEJM 2011;364:248–54. 10.1056/NEJMcp1009570 21247315

[9] Kitson MT, Roberts SK. D-livering the message: the importance of vitamin D status in chronic liver disease. J Hepatol 2012;57:897–909. 10.1016/j.jhep.2012.04.033 22634121

[10] Arteh J, Narra S, Nair S. Prevalence of vitamin D deficiency in chronic liver disease. Dig Dis Sci 2010;55:2624–2628. 10.1007/s10620-009-1069-9 19960254

[11] Stokes CS, Volmer DA, Grünhage F, Lammert F. Vitamin D in chronic liver disease. Liver Int. 2013;33:338–352. 10.1111/liv.12106 23402606

[12] Cervoni JP, Thévenot T, Weil D, Muel E, Barbot O, Sheppard F, et al C-reactive protein predicts short-term mortality in patients with cirrhosis. J Hepatol. 2012;56:1299–1304. 10.1016/j.jhep.2011.12.030 22314431

[13] Waidmann O, Brunner F, Herrmann E, Zeuzem S, Piiper A, Kronenberger B. Macrophage activation is a prognostic parameter for variceal bleeding and overall survival in patients with liver cirrhosis. J Hepatol. 2013;58:956–961. 10.1016/j.jhep.2013.01.005 23333526

[14] Trépo E, Ouziel R, Pradat P, Momozawa Y, Quertinmont E, Gervy C, et al Marked 25-hydroxvitamin D deficiency is associated with poor prognosis in patients with alcoholic liver disease. J Hepatol 2013;59:344–350. 10.1016/j.jhep.2013.03.024 23557869

[15] Stokes CS, Krawczyk M, Reichel C, Lammert F, Grünhage F. Vitamin D deficiency is associated with mortality in patients with advanced liver cirrhosis. Eur J Clin Invest. 2013 11 15. [Epub ahead of print]. 10.1111/eci.12205 24236541

[16] Fernández J, Acevedo J, Castro M, Garcia O, de Lope CR, Roca D, et al Prevalence and risk factors of infections by multiresistant bacteria in cirrhosis: a prospective study. Hepatology. 2012;55:1551–1561. 10.1002/hep.25532 22183941

[17] European Association for the Study of the Liver. EASL clinical practice guidelines on the management of ascites, spontaneous bacterial peritonitis, and hepatorenal syndrome in cirrhosis. J Hepatol. 2010;53:397–417. 10.1016/j.jhep.2010.05.004 20633946

[18] McShane LM, Altman DG, Sauerbrei W, Taube SE, Gion M, Clark GM. Statistics Subcommittee of the NCI-EORTC Working Group on Cancer Diagnostics. Reporting recommendations for tumor marker prognostic studies (REMARK). J Natl Cancer Inst 2005;97:1180–1184. 16106022 10.1093/jnci/dji237

[19] Finkelmeier F, Kronenberger B, Köberle V, Bojunga J, Zeuzem S, Trojan J, et al Severe 25-hydroxyvitamin D deficiency identifies a poor prognosis in patients with hepatocellular carcinoma—a prospective cohort study. Aliment Pharmacol Ther. 2014;39:1204–1212. 10.1111/apt.12731 24684435

[20] García-Álvarez M, Pineda-Tenor D, Jiménez-Sousa MA, Fernandez-Rodriguez A, Guzman-Fulgencio M, Resino S. Relationship of vitamin D status with advanced liver fibrosis and response to hepatitis C virus therapy: A meta-analysis. Hepatology. 2014 6 27. [Epub ahead of print]. 10.1002/hep.27281 24975775

[21] Guzmán-Fulgencio M, García-Álvarez M, Berenguer J, Jiminez-Sousa MA, Cosin J, Pineda-Tenor D, et al Vitamin D deficiency is associated with severity of liver disease in HIV/HCV coinfected patients. J Infect. 2014;68:176–184. 10.1016/j.jinf.2013.10.011 24184809

[22] Malham M, Jørgensen SP, Ott P, Agnholt J, Vilstrup H, Borre M, et al Vitamin D deficiency in cirrhosis relates to liver dysfunction rather than aetiology. World J Gastroenterol. 2011;17:922–925. 10.3748/wjg.v17.i7.922 21412501 PMC3051142

[23] Putz-Bankuti C, Pilz S, Stojakovic T, Scharnagl H, Pieber TR, Trauner M, et al Association of 25-hydroxyvitamin D levels with liver dysfunction and mortality in chronic liver disease. Liver Int. 2012;32:845–851. 10.1111/j.1478-3231.2011.02735.x 22222013

[24] Zhang C, Zhao L, Ma L, Lv C, Ding Y, Xia T, et al Vitamin D status and expression of vitamin D receptor and LL-37 in patients with spontaneous bacterial peritonitis. Dig Dis Sci 2012;57:182–188. 10.1007/s10620-011-1824-6 21755299

[25] Jalan R, Fernandez J, Wiest R, Schnabl B, Moreau R, Angeli P, et al Bacterial infections in cirrhosis: a position statement based on the EASL Special Conference 2013. J Hepatol. 2014;60:1310–1324. 10.1016/j.jhep.2014.01.024 24530646

[26] Han YP, Kong M, Zheng S, Ren Y, Zhu L, Shi H, et al Vitamin D in liver diseases: from mechanisms to clinical trials. J Gastroenterol Hepatol. 2013;28:49–55. 10.1111/jgh.12016 23855296

[27] Almerighi C, Sinistro A, Cavazza A, Ciaprini C, Rocchi G, Bergamini A. 1Alpha,25-dihydroxyvitamin D3 inhibits CD40L-induced pro-inflammatory and immunomodulatory activity in human monocytes. Cytokine 2009;45:190–197. 10.1016/j.cyto.2008.12.009 19186073

[28] Brenner C, Galluzzi L, Kepp O, Kroemer G. Decoding cell death signals in liver inflammation. J Hepatol. 2013;59:583–594. 10.1016/j.jhep.2013.03.033 23567086

[29] Zhu Y, Mahon BD, Froicu M, Cantorna MT. Calcium and 1 alpha,25- dihydroxyvitamin D3 target the TNF-alpha pathway to suppress experimental inflammatory bowel disease. Eur J Immunol 2005;35:217–224. 15593122 10.1002/eji.200425491

[30] Lemire JM. Immunomodulatory role of 1,25-dihydroxyvitamin D3. J Cell Biochem 1992;49:26–31. 1644850 10.1002/jcb.240490106

[31] Müller K, Haahr PM, Diamant M, Rieneck K, Kharazmi A, Bendtzen K. 1,25-Dihydroxyvitamin D3 inhibits cytokine production by human blood monocytes at the post-transcriptional level. Cytokine 1992;4:506–512. 1337987 10.1016/1043-4666(92)90012-g

[32] Plessis JD, Vanheel H, Janssen CE, Roos L, Slavik T, Stivaktas PI, et al Activated intestinal macrophages in patients with cirrhosis release NO and IL-6 that may disrupt intestinal barrier function. J Hepatol 2013;58:1125–1132. 10.1016/j.jhep.2013.01.038 23402745

[33] Assa A, Vong L, Pinnell LJ, Avitzur N, Johnson-Henry KC, Sherman PM. Vitamin D Deficiency Promotes Epithelial Barrier Dysfunction and Intestinal Inflammation. J Infect Dis. 2014;210:1296–1305. 10.1093/infdis/jiu235 24755435

[34] Fisher L, Fisher A. Vitamin D and parathyroid hormone in outpatients with noncholestatic chronic liver disease. Clin Gastroenterol Hepatol 2007;5:513–520. 17222588 10.1016/j.cgh.2006.10.015

[35] Chen CC, Wang SS, Jeng FS, Lee SD. Metabolic bone disease of liver cirrhosis: is it parallel to the clinical severity of cirrhosis? J Gastroenterol Hepatol 1996;11:417–421. 8743912 10.1111/j.1440-1746.1996.tb00284.x

